# Effect of Phytic Acid Addition on the Structure of Collagen-Hyaluronic Acid Composite Gel

**DOI:** 10.3390/gels9120963

**Published:** 2023-12-08

**Authors:** Yuliya Nashchekina, Evgeny Guryanov, Alexey Lihachev, Gleb Vaganov, Elena Popova, Natalya Mikhailova, Alexey Nashchekin

**Affiliations:** 1 Center of Cell Technologies, Institute of Cytology of the Russian Academy of Sciences, Tikhoretsky Pr. 4, 194064 St. Petersburg, Russia; murlok2000@mail.ru (E.G.); natmik@mail.ru (N.M.); 2 Laboratory «Characterization of Materials and Structures of Solid State Electronics», Ioffe Institute, Polytekhnicheskaya St. 26, 194021 St. Petersburg, Russia; lihachev@mail.ioffe.ru (A.L.); nashchekin@mail.ioffe.ru (A.N.); 3 Institute of Macromolecular Compounds of Russian Academy of Sciences, V.O., Bol’shoy Pr. 31, 199004 St. Petersburg, Russia; glebvaganov@mail.ru (G.V.); men682003@mail.ru (E.P.)

**Keywords:** collagen, hyaluronic acid, phytic acid, composite hydrogels, mesenchymal stem cells

## Abstract

Composite collagen gels with hyaluronic acid are developed tissue-engineered structures for filling and regeneration of defects in various organs and tissues. For the first time, phytic acid was used to increase the stability and improve the mechanical properties of collagen gels with hyaluronic acid. Phytic acid is a promising cross-linker for collagen hydrogels and is a plant-derived antioxidant found in rich sources of beans, grains, and oilseeds. Phytic acid has several benefits due to its antioxidant, anticancer, and antitumor properties. In this work, studies were carried out on the kinetics of the self-assembly of collagen molecules in the presence of phytic and hyaluronic acids. It was shown that both of these acids do not lead to collagen self-assembly. Scanning electron microscopy showed that in the presence of phytic and hyaluronic acids, the collagen fibrils had a native structure, and the FTIR method confirmed the chemical cross-links between the collagen fibrils. DSC and rheological studies demonstrated that adding the phytic acid improved the stability and modulus of elasticity of the collagen gel. The presence of hyaluronic acid in the collagen gel slightly reduced the effect of phytic acid. The presence of phytic acid in the collagen gel improved the stability of the scaffold, but, after 1 week of cultivation, slightly reduced the viability of mesenchymal stromal cells cultured in the gel. The collagen type I gel with hyaluronic and phytic acids can be used to replace tissue defects, especially after the removal of cancerous tumors.

## 1. Introduction

Injuries and surgical operations lead to the loss of volume and functions of various organs and tissues. To replenish the lost tissue volume at the site of injury, tissue-engineered structures based on natural or synthetic polymers are worn [1,2,3]. In general, smooth hydrogels that can conform to the shape of wounds are ideal for abrupt changes in fracture type [4,5]. For hydrogels, there is currently a wide range of different polymeric materials of natural and synthetic origin, such as alginate, chitosan, polymethyl methacrylate, polyethylene glycol, etc. [6,7]. Of particular interest are gels based on extracellular matrix proteins [8,9]. The main extracellular matrix structural component is collagen type I (Col). Currently, gels based on type I collagen are widely used to solve tissue engineering tasks [10,11]. For example, collagen-based scaffolds have osteogenic properties and can be used to regenerate bone tissue [12]. Scaffolds containing collagen are also used for the reconstruction of nerve tissue. Thus, Li et al. improved the efficient process of differentiation of MSCs into neuronal and cellular expression; these are neuronal phenotypes on collagen scaffolds [13]. Also, Park et al. demonstrated therapeutic factors (e.g., neurotrophic factors) with umbilical cord blood cells cultured in collagen gel [14]. Collagen hydrogel in combination with hyaluronic acid is promising for use in the regeneration of different types of tissues. Hyaluronic acid (HA) is a major component of the extracellular matrix and the simplest glycosaminoglycan. HA actively influences cell proliferation and coagulation, as well as tissue remodeling throughout the wound-healing process [15,16]. The collagen and hyaluronic acid hydrogels’ biophysical properties, such as internal microstructure, mechanical strength, swelling rate, and transport of young molecules, are usually determined by fabrication methods. Thus, mixed hydrogels define the relationship between the biophysical manifestations of the gel and its ECM composition. Due to the presence of hyaluronic acid in the scaffold, its swelling coefficient is significantly greater than that of scaffolds based on pure collagen. HA can improve the swelling characteristics of such a hydrogel, primarily due to its high water absorption capacity.

It is known that collagen molecules can form fibrillar structures as a three-dimensional hydrogel network without chemical cross-linkers. If one adds the HA to the collagen solution before polymerization, it will be retained in the fibrillar collagen structure. Hydrogels containing the high molecular weight HA (1.5–1.8 MDa) and collagen I and II mixture have shown that HA does not suppress gelation [17]. When using hyaluronic acid with a low molecular weight, the formation of collagen fibrils was also not disrupted [18]. This hydrogel system provides chondrogenic differentiation and the growth of mesenchymal stromal cells. In addition to the substances contained in the basis of extracellular matrix proteins, it also has disadvantages such as poorly retained form and high degradation rate. This study examined the stability of gels based on collagen and hyaluronic acid. Due to its good solubility in water, hyaluronic acid can be quite easily desorbed from the collagen gel, so it is important to create a strong collagen network that would prevent hyaluronic acid molecules from leaving the composite gel.

Many factors, including the control of molecular weight and modification of hyaluronic acid, can be applied to modulate the properties of Col and HA hydrogel without cross-linkers. Xin et al. found that the addition of low molecular weight HA (155 kDa) enlarged the Col/HA gels elastic modulus; using low molecular weight HA (1.2 MDa) does not induce the opposite effect [19]. It was also found the low molecular weight HA was combined with the collagen network and covered the collagen fibrils. However, low molecular weight HA can be easily desorbed from collagen gel during culture in silent cells. In another study, the addition of low molecular weight HA led to increased gel stiffness, average pore size, and average radius of collagen matrices [20]. The increase in pore size may be related to the swelling caused by HA [21] since HA associated with collagen fibers during fibrillogenesis may also enlarge the fiber radius.

The stability of such hydrogels can be increased, and the desorption of HA can be reduced by obtaining a cross-linked collagen network. Cross-linking agents such as carbodiimide are widely used [22]. Other authors used PEG ester tetrasuccinimidyl glutarate for cross-linking, which interacts with collagen amino groups to produce a stable semi-permeable network hydrogel to survive the chondrocytes [23]. The treatment uses mild conditions that include biologically active factors and cell encapsulation during gel formation. In another case, riboflavin was used to photo-cross-link collagen [24]. The hydrogel was gentler and slowed down enzyme-induced degradation compared to physically cross-linked collagen hydrogel. Thus, covalent collagen cross-linking can assemble a stable network with controlled degradation rates and mechanical mechanisms, and the HA incorporation into different forms determines the biocompatibility of such hydrogels.

Phytic acid (PA) is a promising cross-linking agent for collagen hydrogels. Phytic acid (myoinositol hexalisphosphate, IP6) is a plant-derived antioxidant found in abundant sources of beans, grains, oilseeds, spores, pollen, and organic soil [25]. PA has a number of benefits due to its antioxidant and antitumor properties. In vivo and in vitro experiments have shown the therapeutic effect of phytic acid dephosphorylation in colorectal cancer, colon cancer, and rectal carcinoma [26,27].

PA consists of a myoinositol ring coupled with six phosphate groups via ester bonds [28]. Based on the electrovalent (hydrogen) bonding, anions (O^−^) of PA can bond to the cations of natural polymer. Collagen is also a natural polymer, which includes a sufficient number of amino groups. PA as a cross-linking agent is used to improve the mechanical properties and stability of decellularized tissues. Thus, in the work of Wang et al., it was shown that phytic acid is a perspective biological cross-linking agent, due to its excellent cross-linking effects, resistance to enzymatic degradation, cytocompatibility, and anti-calcification effects [29].

PA is also widely used in dentistry. It was shown in [30], that PA has a positive effect not only on dentin and tooth enamel but also on soft tissues. Despite a number of advantages of phytic acid and its use for stabilizing decellularized tissues, it has not yet found widespread use as a cross-linking agent for collagen scaffolds. However, the work of Tu et al. demonstrated that PA has a cross-linking effect on collagen molecules and does not interfere with the formation of fibrils with native periodicity [31]. However, for practical use in regenerative medicine, it is not so much collagen solutions with cross-linked collagen fibrils that are of particular value, but rather stable hydrogels, which, due to their viscosity, allow them to be fixed in a specific location of a damaged organ or tissue. Hydrogels are widely used in medical applications as scaffolds for tissue engineering and wound healing applications because of their ability to imitate native tissue characteristics [32]. Hydrogels can form tridimensional cross-linked hydrophilic structures by chemical or physical bonds, characterized by possessing highly porous and polymeric interconnected networks that can swell, absorbing large amounts of water without decomposing their polymeric structure.

Collagen-based gels are especially attractive for these purposes, including those with the addition of such an important component of the extracellular matrix as HA. However, due to its good solubility in water, HA, when physically mixed with collagen, is easily desorbed from the gel. Therefore, additional methods for its fixation in collagen gel are required. PA is a potential cross-linking agent for such a two-component gel. At the same time, there is no data in the literature on the cross-linking of collagen with PA in the presence of HA, and there is also no data on the effect of HA on the ability of PA to cross-link collagen molecules.

This study aimed to obtain a stable hydrogel based on collagen and HA cross-linked with PA, and studied the influence of each component on the structural, mechanical, and biological properties on properties of the hydrogel.

## 2. Results and Discussion

### 2.1. Study of Phytic and Hyaluronic Acids on the Kinetics of Collagen Self-Assembly by UV Spectroscopy

**Figure 1:**
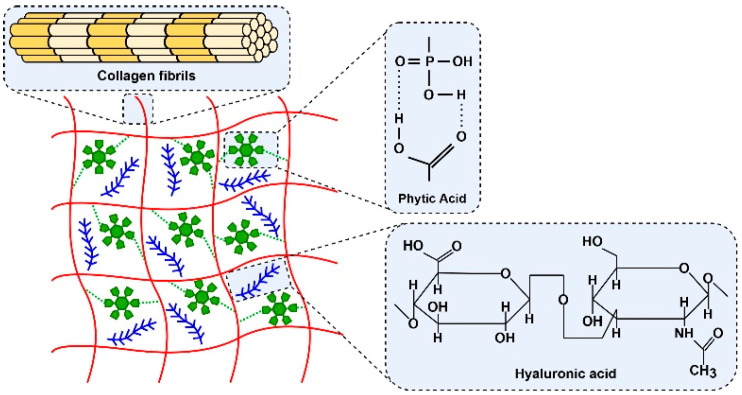
Schematic representation of composite HA gel in collagen gel cross-linked with PA.

Composite gels based on collagen and HA mimic extracellular matrices. However, due to the good solubility of HA in aqueous media, it must be fixed in the fibrils of the collagen gel (Figure 1).

**Figure 2:**
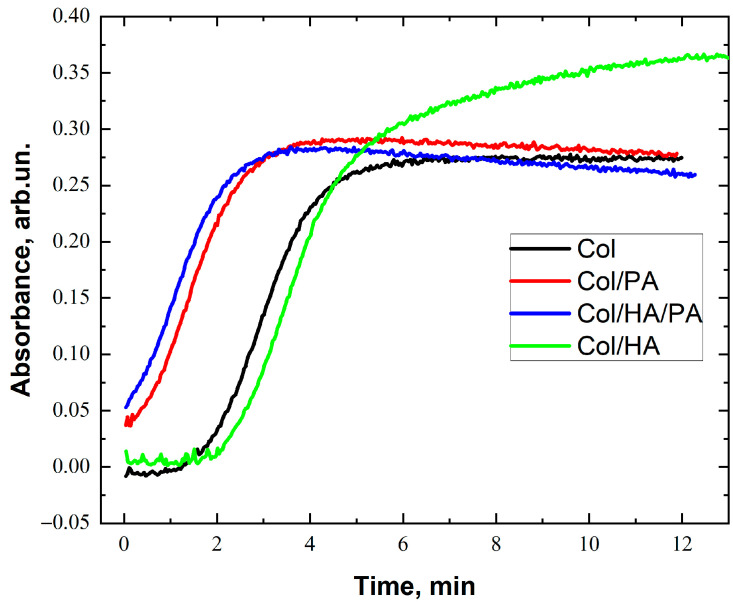
The phytic acid effect on the self-assembly kinetics of collagen (0.5 mg/mL in PBS) was incubated at 37 °C (absorbance at 310 nm).

To influence the kinetics of collagen self-assembly, optical transmittance at 310 nm was measured for 5 min with a collagen concentration of 0.5 mg/mL in solution, and PA was measured at a concentration of 0.2%. Earlier in this article, data were reported on the study of medical PA wires during collagen self-assembly in vitro [31]. It was shown that small concentrations of PA in collagen dissolution up to 1% caused attraction to the protein mass. To prevent protein self-assembly, further increasing the efficiency of PA in dissolution not only does not promote collagen self-assembly but leads to inhibition of fibril formation. In our work, we chose a phytic acid content in the collagen solution of 0.2% solute to solution volume, which corresponded to 0.001% solute to protein mass. The obtained data are presented in Figure 2.

The process of the self-assembly of collagen in vitro, according to the nucleation-propagation mechanism, includes three main phases: a short delay phase when optical transparency slowly decreases and then transparency drops quickly, a growth phase, and a plateau phase when the transparency plateau reaches a minimal equilibrium level [33]. As shown in Figure 2, the self-assembly of collagen in the body, compared with biological collagen, had a pronounced sigmoidal growth curve (including a slight deceleration phase, a growth phase, and a plateau phase). In addition, it was possible to constantly monitor changes in the time of lag phases and the time to occur at the equilibrium level of collagen self-assembly with and without PA. These data allowed one to propose that the kinetics of collagen self-assembly may be accelerated. The presence of HA in collagen dissolution did not affect the process of collagen self-assembly, which ensured the simultaneous initiation of the process of fibril formation and its continuation. The obtained data on the absence of HA in the process of collagen fibril formation have previously been demonstrated in other works [17].

The collagen self-assembly process was thermally controlled, which included electrostatic interaction forces, hydrophobic forces, and hydrogen bonds between polar collagen molecules [34]. Since strong electronegativity caused the release of electronegative oxygen, PA could easily create stable electrovalent bonds with the amino group of collagens in an acidic medium. The stability of the hydrogen bonds was far superior to the observed hydrogen bonding relationship between collagen because the oxygen anions of PA provided independence of electronegativity [30]. So, the kinetics of collagen self-assembly could be accelerated by the formation of hydrogen bonding between PA and the amino group of collagens. In the first step, a large amount of PA was coupled to the amino groups of collagens, resulting in most of the hydrogen bond sites in collagen being occupied by PA. Consequently, repulsion between PA and neighboring collagen molecules would prevent collagen self-assembly and only a minimal amount of HA favors the formation of a collagen meshwork that will retain HA.

**Figure 3:**
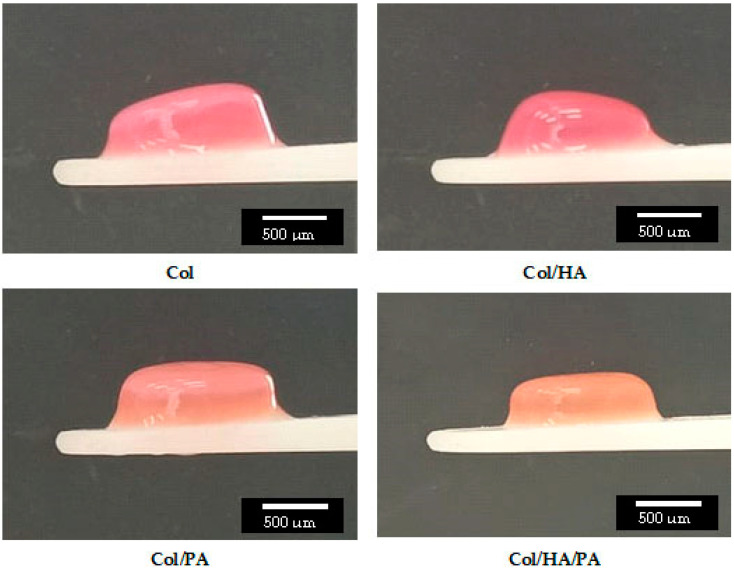
The appearance of the obtained hydrogels.

The appearance of the obtained hydrogels is shown in Figure 3. The diameter of the sample was 1 cm, and the height was 300–500 μm. As can be seen in the figure, hydrogels with phytic acid had better stability compared to hydrogels without phytic acid. Gels with phytic acid had a regular shape and the upper boundary of the hydrogel had clear horizontal boundaries.

### 2.2. FTIR Analysis

**Figure 4:**
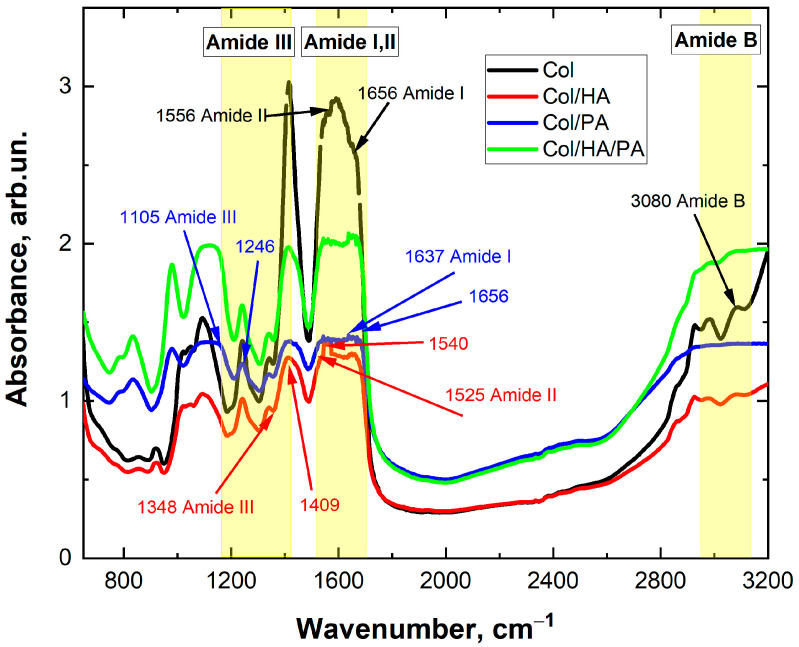
FTIR spectra of collagen without and with phytic and hyaluronic acid.

To evaluate the changes produced by PA cross-linking and HA addition to the collagen solution, as well as the saving of its native structure, the Fourier IR analysis of Col and Col/PA, Col/HA, and Col/HA/PA were provided (Figure 4).

Typically, the IR spectra of collagen samples showed vibrational modes of 1650, 1560, and 1235 cm^−1^, characteristic of amide bands I, II, and III, respectively. The measured FTIR spectrum of the native collagen (Figure 4, black spectrum) showed bands to its specific molecular organization related to the collagen peptide linkages, namely NH and OH stretching: amide B (3080 cm^−1^), amide I (1656 cm^−1^), amide II, and amide III (1556, 1246 cm^−1^) [34,35]. It is important to mention that knowing the amide I peak intensity allowed the evaluation of the conformation of collagen molecules in the native and denatured states [36,37]. The vibrations of amide carbonyl along the carbonyl backbone can be assigned to the amide I band. Amide II consists of amide NH bending vibrations and CN stretching vibrations [38]. These bands can be used to assess the extent to which the triple helical structure of collagen is retained in a given material [39].

The effective cross-linking of PA and Col is the result of bonding anionic O=P–OH groups of PA to cationic NH_2_ groups of Col [26]. The analysis of the Col/PA spectrum (Figure 4, blue spectrum) showed characteristic oscillations of the amide groups of collagens. In addition, characteristic oscillations in phosphate groups of PA appeared at 1105 cm^−1^ (characteristic phosphate radical peak), indicating the successful introduction of PA.

The cross-linking of HA and collagen was also confirmed by measuring FTIR spectra. The obtained spectra showed that the absorption peaks appeared at 1641 cm^−1^, corresponding to amide I (1535 cm^−1^), amide II, and amide III (1242 cm^−1^). They are the characteristic absorption peaks of C=O, N–H, and C–N, respectively.

Figure 4, the green line reflects the overall effect of the components added to the mixture with collagen. The functional groups present in pure Col, Col/HA, Col/PA, and Col/Ha/Pa showed characteristic vibrations for the amide bands of proteins 1637 cm^−1^ and 1656 cm^−1^ (amide I), 1525 cm^−1^ and 1540 cm^−1^ (amide II), and 1348 cm^−1^ and 1409 cm^−1^ (amide III).

When samples containing PA and HA were examined, a peak at 1000 cm^−1^, which corresponds to the peak of the standard emission of HA, was visible, and a peak at 1100 cm^−1^, which corresponds to PA, was also clearly visible [26]. Also, assuming that the defining peak of HA was the peak at 1000 cm^−1^, it can be argued that the crystals without PA did not contain HA, especially when considering the Col/HA and Col/HA/PA samples. Thus, the FTIR results obtained allowed us to assume the chemical cross-links between the collagen and PA in the presence of HA.

### 2.3. Thermal Characterization

**Figure 5:**
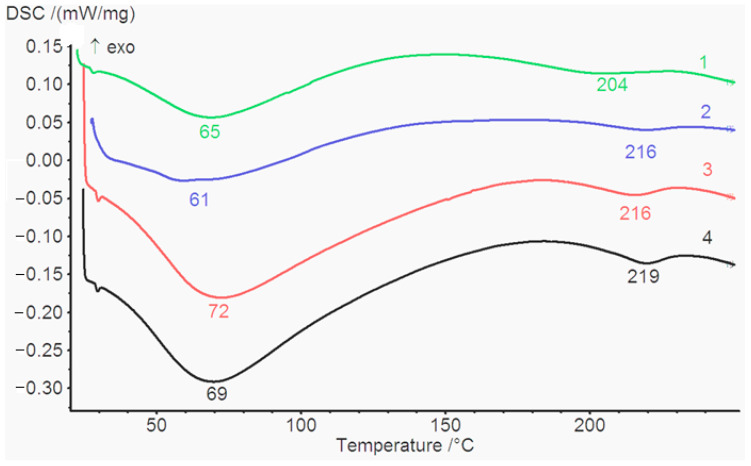
DSC curves for non-cross-linked and cross-linked collagen gels: Col (green)—1, Col/HA (blue)—2, Col/PA (red)—3, and Col/HA/PA (black)—4.

The H bonds formed between the OH and NH_2_ groups of hydroxyproline and glycine in collagen molecule stabilize the triple helical structure. This leads to the formation of a fibrous crystalline zone embedded into an amorphous matrix [39]. Two main transitions are displayed in the temperature range from 20 °C to 250 °C. The endothermic peaks between 20 °C and 120 °C, resulting from dehydration and collagen thermal denaturation (Td), are overlapping. The melting temperature (Tm) (from 205 °C to 235 °C) is related to the cross-linking degree of collagen gels and the degradation of these materials. Collagen stability can be evaluated by Td and Tm. The Td expresses the transition temperature of the collagen molecule conformation from triple helix to the static coil and can be exactly determined by DSC The maximum temperature in the DSC curves reflects the Td and gives indications of the intra and intermolecular interactions in the sense that if they are inhibited, Td has low values and the pronounced cross-linking leads to a raise these values [40]. The DSC curves of Col, Col/HA, Col/PA, and Col/HA/PA hydrogel are presented in Figure 5. The dehydration temperature of each gel is indicated by the corresponding endothermic peaks. Hydroxyproline plays a major role in the stabilization of the collagen triple-helix structure due to its hydrogen-bonding ability through its –OH group [41]. The temperature feature of the dehydration values calculated from the DSC measurements is presented in Figure 5. The DSC results showed that the cross-linked PA hydrogel exhibited an elevated dehydration temperature. The dehydration temperature for collagen gel increased from 65 °C (Col) to 72 °C (Col/PA), and from 61 °C (Col/HA) to 69 °C. The presence of HA reduced the Td for both the collagen gel and collagen gel cross-linked with PA. The hydroxyl groups of HA compete for the amino groups of collagens together with the hydroxyl groups of PA. The cross-linking ability of PA in relation to collagen was confirmed using the DSC method in the work Tu [31]. The authors of this study demonstrated an increase in Td. The difference in T values for the collagen-based sample alone seems to be due to the fact that in our study we used collagen obtained by acid extraction. This method allows the preservation of the terminal telopeptides, which, among other things, are involved in the process of fibrillation and gel formation. The authors of the above article did not provide data on the properties of the collagen used. However, the source of collagen, namely the tendons of cattle, allowed us to assume that this collagen was obtained by enzymatic extraction, which leads to partial damage to the terminal telopeptides.

The analysis of the melting curves obtained by DSC revealed that the Tm of collagen gel after adding HA and PA increased. The Tm for the collagen gel increased from 204 °C (Col) to 216 °C, 216 °C, and 219 °C for Col/HA, Col/PA, and Col/HA/PA respectively. After the addition of PA, the Tm of Col/PA and Col/HA/PA were higher than for Col, suggesting an increase in the collagen cross-linking degree upon the treatment. The result showed that the treatment method could strengthen the crystalline zone stability of the collagen gel. The temperature cycle experiment showed that transition disappears in the second cycle.

**Figure 6:**
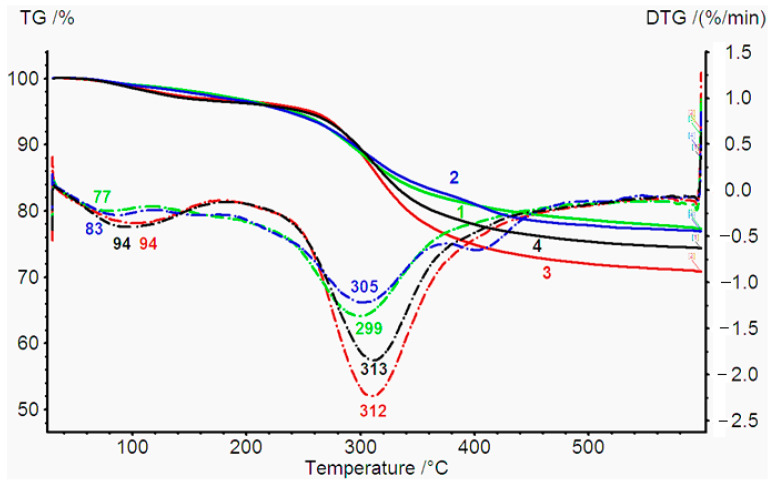
Thermogravimetric (TGA) analysis of the hydrogel Col (green)—1, Col/HA (blue)—2, Col/PA (red)—3 and Col/HA/PA (black)—4. TG/% = TG/Mass %.

TGA was used to analyze the collagen hydrogel thermo stability. Figure 6 represents the pyrolytic pattern of Col, Col/HA, Col/PA, and Col/HA/PA hydrogel. The entire weight loss process was realized in three phases. The first phase is related to the structural water loss (dehydration) of the gel (50–200 °C). All the hydrogels demonstrated a weight loss due to the loss of free water. It should be noted that water loss for all samples occurred at different temperatures. First of all, water left the sample with HA. Indeed, it is known that HA captures water very well. The binding strength of such water with HA was less than that with collagen. Moreover, the temperature T value for the endothermic peak of the sample with HA (77 °C) corresponded to the literature data for a gel-based only on HA (78 °C) [42]. The hydration temperature of collagen hydrogel increased by 6 °C and was 83 °C. In the presence of PA, the dehydration temperature of the samples increased by 11 °C. Thus, according to the TGA data, it can be concluded that PA contributed to the binding of water with collagen hydrogels. Perhaps this is due to the fact that it is more difficult for water to escape from the collagen fibrils stitched with phytic acid. For samples containing PA (samples 3 and 4) in the region of 150–160 °C, we observed an exothermic peak. According to the literature data, the temperature of 150 °C corresponds to the temperature at the beginning of the denaturation of PA [43]. It should be noted that the introduction of phytic acid into the collagen hydrogel increased the denaturation temperature of collagen. The denaturation temperature of collagen after the addition of PA increased from 305 °C to 313 °C. The introduction of HA into the collagen gel, on the contrary, reduced the denaturation temperature from 305 °C to a delivery of 299 °C. However, the introduction of PA to the collagen gel with HA contributed to an increase in the stability of the collagen gel, which led to an increase in the destruction temperature of the hydrogel from 299 °C to 312 °C. The third phase demonstrated the carbonization of material (300–600 °C) [43]. This fact means that the cross-linking of PA leads to structural changes and may possess a stronger interaction between collagen molecules, thus, resulting in enlarged thermal stability of the scaffold before a temperature of 150 °C. It is the temperature of thermal decomposition because of the carbonization of the sample and the removal from the PA of the OH groups.

### 2.4. Viscoelastic Properties

**Figure 7:**
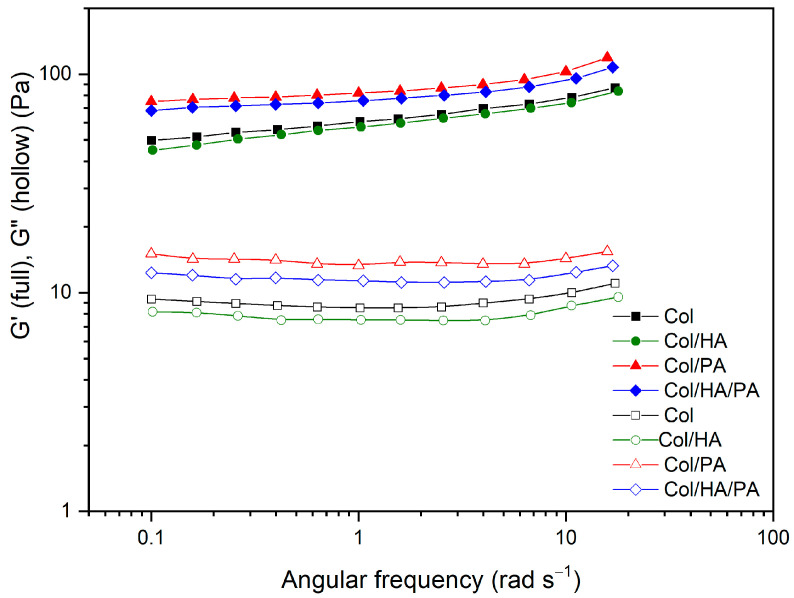
Elastic (G′) and viscous (G″) moduli as a function angular frequency of collagen hydrogels obtained with phytic acid and hyaluronic acid.

The viscoelastic properties of hydrogels obtained from pure collagen (Col) and collagen after adding hyaluronic acid (Col/HA) or phytic acid (Col/PA) and (Col/HA/PA) were analyzed by an oscillating rheological test, and the frequency dependence curves of G′ (elastic modulus) and G″ (viscous modulus) moduli of these hydrogels were recorded. As can be seen from Figure 7, the G′ value was always higher than the corresponding G″, suggesting that these systems had a gel-like structure [44]. Adding PA to collagen helped increase both G′ and G″. The addition of PA to collagen increased the elasticity of the system, as evidenced by the increase in G′ shown in the figure. Since the elastic behavior of the hydrogel depends on the number of cross-links, the increase in the shear elastic modulus of the gel is possibly due to the processes of the appearance of additional cross-links of collagen with PA. A similar process was shown in the work [45].

The addition of hyaluronic acid slightly reduced both G′ and G″.

### 2.5. In Vitro Degradation

**Figure 8:**
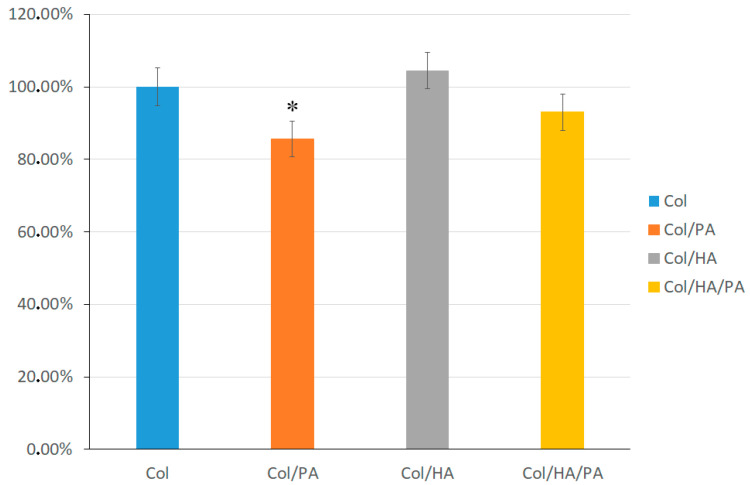
In vitro degradation of the Col hydrogel without and with PA. (* *p* < 0.05).

The degradation of the collagen hydrogel before and after the addition of PA was evaluated using a hydroxyproline assay. Figure 8 shows that slightly more hydroxyproline was desorbed from the hydrogel with HA after 1 week of incubation in the phosphate buffer compared to the Col hydrogel. However, these values were within the measurement error, so it is not worth arguing that HA affects the degradation rate of collagen gel. A significant decrease in the amount of hydroxyproline in the phosphate buffer after incubation was observed in samples with PA. A decrease in the amount of hydroxyproline in the phosphate buffer was observed both in the collagen-only sample and the collagen- and HA-based sample. These results indicated that the incorporation of amino acids was necessary to enhance biostability.

### 2.6. Scanning Electron Microscopy

**Figure 9:**
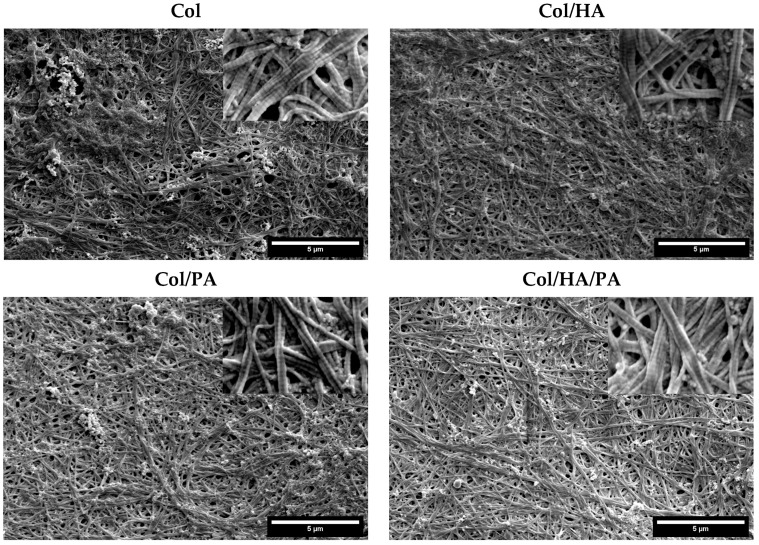
SEM images of collagen fibrils obtained with phytic acid and hyaluronic acid (all insets—20 times magnified areas of corresponding samples).

The effect of the composition of phytic and hyaluronic acid on collagen fibrils was investigated by SEM. The SEM images are presented in Figure 9 and show that collagen fibrils of all tested groups were woven into an assembled structure. These facts confirmed the self-assembly property of collagen was manifested in the formation of phytic and hyaluronic acids. The SEM data demonstrated that both phytic and hyaluronic acid did not affect the change in fibril diameter. Previously, it was shown in the literature that in the presence of phytic acid, finer fibrils are formed compared to collagen fibrils without phytic acid [31]. In our study, we did not observe such a dependence. This is due to the fact that the concentration of phytic acid in the collagen solution was too low to have a significant effect on the diameter of the forming fibrils. Indeed, a study by Tu et al. showed that the diameter of collagen fibrils decreased with an increasing phytic acid concentration. The presence of HA also did not affect the diameter of collagen fibrils. Collagen monomers assemble into fibrils not only in the longitudinal but also in the transverse direction [46]. In the presence of PA or HA, the likelihood of collision between phytic/hyaluronic acid and collagen molecules may be increased, leading to an increase in the number of nucleation sites and increased competition between nuclei for collagen molecules [47].

Natural D-periodicity is an important feature of the nativeness of collagen fibrils [46]. This natural D-periodicity of collagen has a great influence on the mechanical and biological characteristics of collagen scaffolds [48,49,50]. The microtopology of collagen fibrils was also assessed using SEM. As shown in Figure 9, fibrils in all tested groups had obvious D-periodicity. These results showed that the coexistence of phytic acid, hyaluronic acid, and collagen did not affect the characteristic periodicity of collagen fibrils.

**Figure 10:**
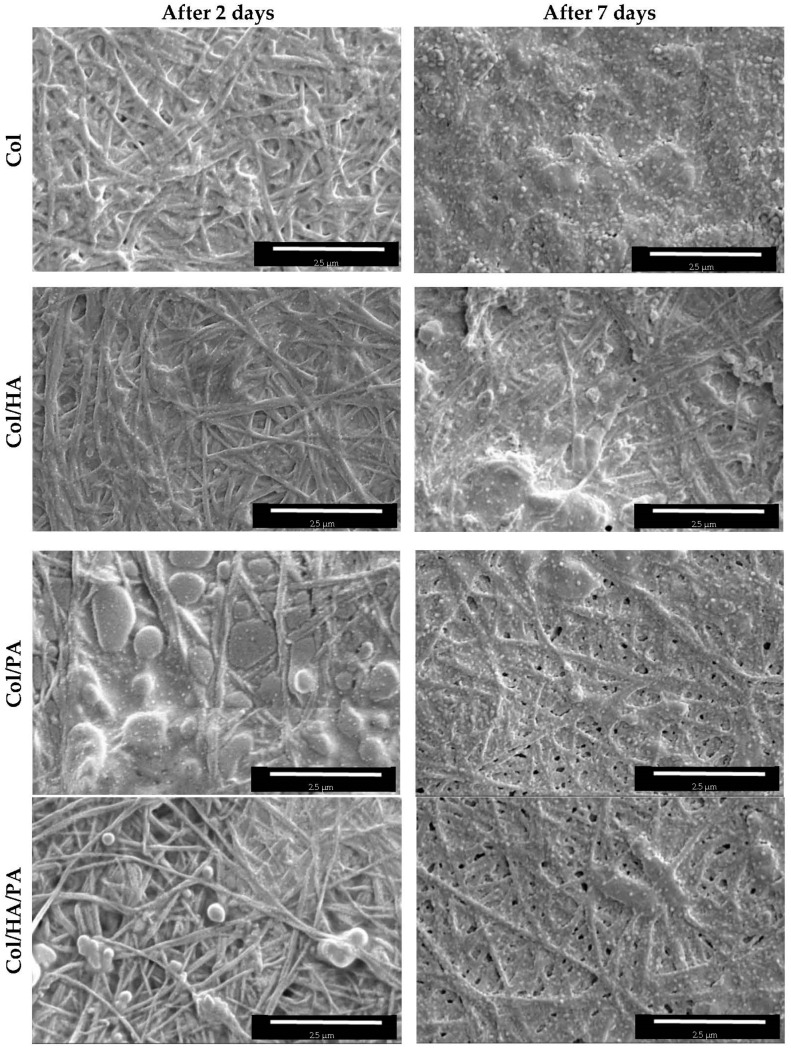
Comparison of SEM images in relation to specimen types and degradation time.

SEM results showed that the PA presence in collagen gel did not determine the native structure of collagen fibrils. It was also shown that the native structure of PA cross-linked collagen fibrils did not degrade after 2 days of incubation in constant phosphate buffer conditions and 7 days of incubation in constant conditions. Differently, collagen gels without modification retained their native structure after 2 days of incubation and were almost completely stationary after 7 days of incubation under consistent conditions (Figure 10).

### 2.7. Cells Interaction with Gels

Both collagen and HA play an important role in the development and maintenance of the extracellular matrix and also realize the biochemical signals to cells to perform their functions: adhesion, proliferation, and differentiation of extracellular matrix proteins [51].

**Figure 11:**
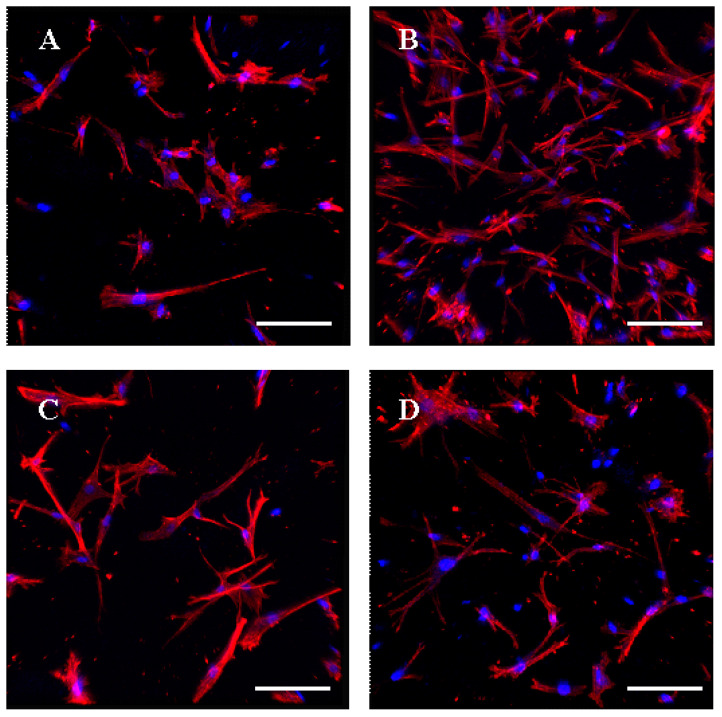
Confocal microscopy of fetMSC cultured in collagen gels for 2 days. (**A**)—collagen gel, (**B**)—collagen gel with phytic acid, (**C**)—collagen gel with hyaluronic acid, (**D**)—collagen gel with hyaluronic acid and phytic acid. Scale bar 100 µm.

When analyzing cells in the gel after 2 days of cultivation under a confocal microscope (Olympus FV3000, Japan) (Figure 11), it could be seen that the cells were evenly distributed throughout the collagen gel. In a pure collagen gel, they had an elongated spindle shape, characteristic of MSC cells, not a cell at all (Figure 11A). It should be noted that the highest number of cells was visualized in the image of the collagen gel cross-linked with phytic acid (Figure 11B). When hyaluronic acid was added, cells cultured in such a composite gel acquired grown cells or phyllopodia (Figure 11C). The number of cells and the number of phyllopodia or grown cells were visualized in the image of the composite gel with hyaluronic acid and phytic acid cross-linked (Figure 11D). Based on the results obtained, it can be concluded that phytic acid had no toxic effects on the human body, while hyaluronic acid affected the morphology of cells cultured in such gels.

**Figure 12:**
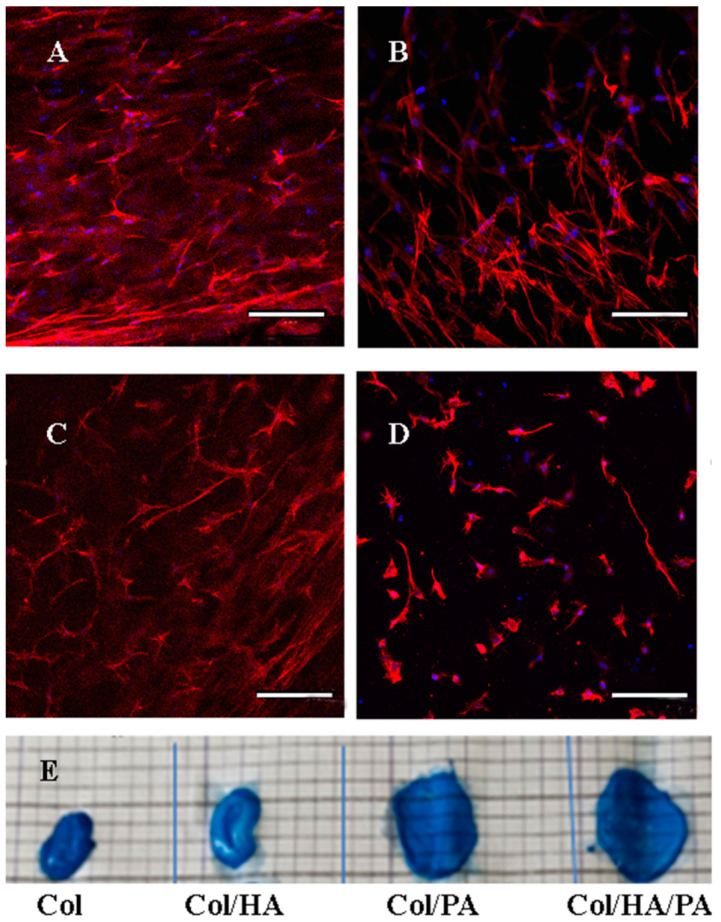
Confocal microscopy of fetMSC cultured in collagen gels for 7 days. (**A**)—collagen gel, (**B**)—collagen gel with phytic acid, (**C**)—collagen gel with hyaluronic acid, (**D**)—collagen gel with hyaluronic acid and phytic acid, (**E**)—Photo of gels with fetMSC after 7 days of cultivation. Scale bar 100 µm.

Cells in the gel after 7 days of cultivation were also stained and analyzed using a confocal microscope (Figure 12A–D). Cells in all gels actively proliferated and formed a dense network of actin filaments, but their number in the gel with hyaluronic acid and phytic acid (Figure 12D) was inferior to the number of cells in the other three samples (Figure 12A–C). In the future, we plan to study this effect in more detail.

When analyzing the external shape of gels with cells after 7 days of cultivation, it was found that gels without phytic acid contracted (Figure 12E). It is known from the literature data that cells in the process of active proliferation are able to “compress” gels, which leads to their contraction or a decrease in volume [52]. Moreover, in the process of contraction, the vital functions of cells cultured inside such gels deteriorated. Cross-linking of gels increased their rigidity and prevented the contraction of gels by cells. The obtained results allow us to assume that collagen gels cross-linked with phytic acid have increased rigidity compared to the control sample, collagen gels without the addition of phytic acid.

### 2.8. MTT-Analysis

**Figure 13:**
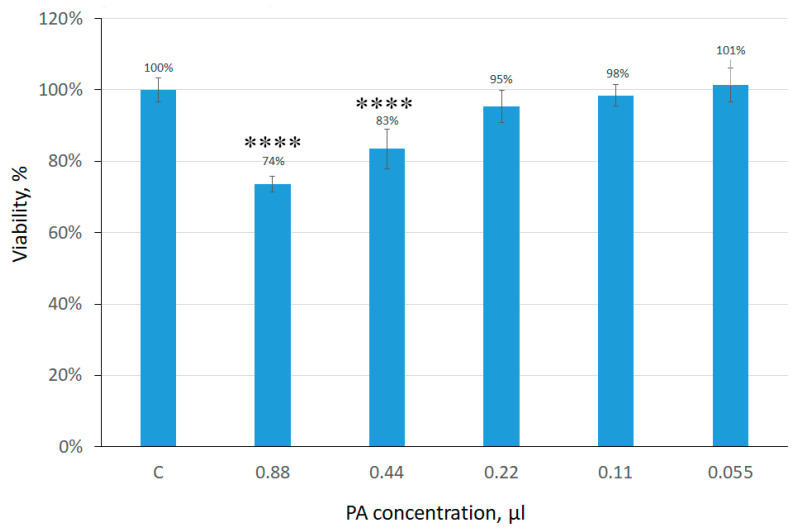
MTT assay of FetMSCs (**** *p* < 0.0001).

**Figure 14:**
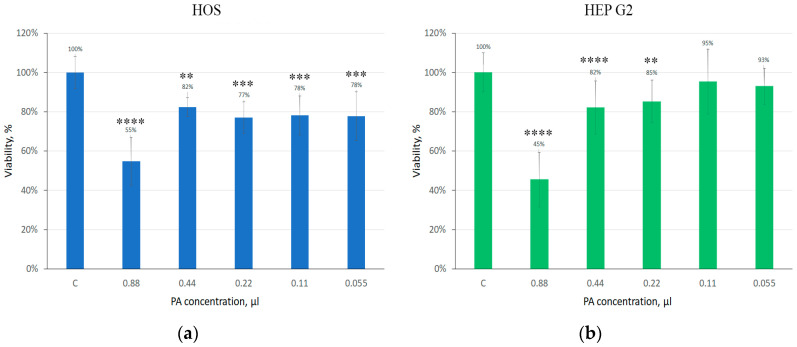
MTT assay of Hos (**a**) and HepG2 (**b**). (**** *p* < 0.0001, *** *p* < 0.001, ** *p* < 0.01).

Figure 13 shows that an increase in the concentration of phytic acid in the nutrient medium decreased cell viability. Preliminary data showed that in the bound state with collagen, phytic acid did not have a toxic effect on cultured FetMSCs. However, as can be seen from confocal microscopy data, the number of spread cells in the hydrogel decreased after 1 week of cultivation. Based on the data obtained, we can assume that within 1 week phytic acid begins to be released from the collagen gel and affects the viability of cells. As already noted in the Section 1, PA has an inhibitory effect on cancer cells. To confirm this statement, we performed preliminary MTT test experiments to assess the effect of phytic acid on HepG2 (liver carcinoma) and HOS (osteosarcoma) cells. The MTT analysis data on the assessment of cell viability depending on the phytic acid concentration are presented below (Figure 14).

As can be seen from the diagrams, these cell types were more sensitive to PA compared to the normal, unformed FetMSCs line. The obtained results allow us to assume that the formed hydrogels with phytic acid can be used in the tissue area after the removal of cancerous tumors. PA released from the hydrogel during degradation will inhibit the growth of metastases.

The extracellular matrix is a network of macromolecules that provides biomechanical and biochemical cues to the tissue cells. The extracellular matrix is insoluble, which is preliminary due to highly cross-linked extracellular matrix proteins such as collagens [53], and, thus, is a stable source of signaling molecules of the cell. All extracellular matrixes are composed of three types of molecules: proteins, glycoproteins, and proteoglycans. Collagen is the most abundant fibrous protein found in the mammalian extracellular matrix [54]. HA is a unique GAG that does not contain covalently bound core proteins [55]. HA non-covalently binds to aggrecans through link proteins [56]. The high viscosity of HA confers resistance to compressive forces and makes it especially important in load-bearing tissues. HA cell receptors regulate functions including induction of chondrogenesis, osteogenesis, neurogenesis, cardiogenesis, and angiogenesis, increasing proliferation of cells including astrocytes and endothelial cells, and controlling inflammation by binding monocytes [57]. In the body, collagen and HA create semi-interpenetrating networks [58].

Therefore, it is very important to use both components of the extracellular matrix for cultivation and transplantation when forming scaffolds. With a simple physical mixing, hyaluronic acid comes out of such a composite gel very easily. Therefore, as noted above, there are a number of methods for fixing hyaluronic acid in the gel. For the first time, we suggested using phytic acid to form a cross-linked gel that holds hyaluronic acid. Indeed, in some works, the cross-linking ability of phytic acid was previously shown. The cross-linking ability of phytic acid in the presence of hyaluronic acid has not yet been studied. We have shown for the first time that hyaluronic acid does not interfere with the cross-linking of collagen fibrils. These three components, namely type I collagen, hyaluronic acid, and phytic acid form a stable gel.

It was also previously noted that phytic acid inhibits the proliferation of cancer cells. We conducted additional studies to assess the different concentrations of phytic acid on the viability of both healthy tissue cells (MSCs) and liver cancer cell lines (HepG2) and osteosarcoma (HOS). The research results are presented below.

## 3. Conclusions

In summary, we have developed a new composite gel, which, thanks to its composition, namely collagen and hyaluronic acid, was as close as possible to the extracellular matrix. In the body, these two components are firmly connected to each other and actively participate in cellular processes. With conventional in vitro mixing, hyaluronic acid is easily desorbed from such a composite gel; we were able to obtain a stable composite gel by cross-linking collagen fibrils with phytic acid. It was shown that the presence of phytic and hyaluronic acids increased the stability of the gel, but did not affect the native structure of collagen fibrils. The good spatial organization of MSCs cultured inside composite gels during the first few days indicated their biocompatibility. However, despite the presence of a number of publications on the advantages of phytic acid, which has antioxidant and anticancer properties, this issue has not yet been sufficiently studied, so we plan to investigate in the future the effect of phytic acid on the viability of both normal and cancer cells. This will allow us to effectively implant the developed stable composite gel into the area of damage to replace defects after the removal of oncological tumors.

We assume that the hydrogels we have developed may be important for the tasks of regenerative medicine. The resulting three-component gel consisting of type I collagen, hyaluronic acid, and phytic acid in comparison with a single-component gel based on type I collagen has such advantages including:

1. Hyaluronic acid, as noted above, includes the stimulation of chondrogenesis, osteogenesis, angiogenesis, and cardiogenesis, enlarging the proliferation of cells (astrocytes and endothelial cells, and controlling inflammation by binding monocytes).
2. Phytic acid performs two tasks. Firstly, it increased the stability of the gel based on type I collagen and hyaluronic acid. Secondly, it reduced the viability of cancer cells. Moreover, it should be noted that the degree of decrease in the viability of cancer cells was greater compared to the decrease in the viability of normal cells, namely MSCs.
3. A potential practical application of such gels is to fill the volume of tissues after removal of tumors. Such hydrogels can be used both with the patient’s MSCs and without cells. When using gels with the patient’s MSCs, the cells are cultured in vitro for several days. In our study, it was shown that for several days the cells actively proliferated inside the gel. Next, the resulting cellular tissue-engineered structure is transplanted into the area of removal of a cancerous tumor. In the process of degradation, MSCs migrate into the surrounding tissues, the released hyaluronic acid promotes the formation of new vessels, and phytic acid prevents the formation of metastases.

## 4. Materials and Methods

### 4.1. Production of Hydrogels

Collagen was obtained from rat tail tendons by acid extraction. Type I collagen was obtained by acid extraction from rat tail tendons. Collagen was extracted in 0.5 M acetic acid. After the protein was dissolved, the collagen was precipitated with sodium chloride. After precipitation, the collagen was dissolved in a 0.5 M acetic acid solution, and dialysis with water was performed to remove the sodium chloride. Then dialysis was performed with a 0.1 M acetic acid solution. Further deposition of collagen was carried out by dialysis of a collagen solution with a 0.02 M sodium hydrophosphate solution. The resulting precipitate was centrifuged and dissolved in a 0.01% acetic acid solution. The sterilization of the collagen solution was carried out by dialysis with a chloroform solution in the form of a 0.01% acetic acid solution.

A solution of collagen was prepared on ice in an acetic acid 0.01% solution (Reaktiv, St. Petersburg, Russia) at a concentration of 2 mg/mL (Col) [59]. To prepare the collagen gel, the physiological ionic strength and pH of the collagen I solution was adjusted to 7.4 using a 10× medium 199 (Gibco, USA) and NaOH 50% (*w*/*w*) aqueous phytic acid (PA) solution (Sigma-Aldrich, St. Louis, MO, USA). To cross-link collagen during the preparation of the gel, phytic acid was added to the protein solution in an amount of 0.2% of the volume of dissolved collagen (Col/PA). To obtain composite gels, hyaluronic acid (HA) (sodium salt from Streptococcus equi, mol. wt. 1.5–1.8 × 10^6^ Da (Sigma, USA)) was added to the collagen solution before gelling in an amount of 5% by weight of collagen (Col/HA). To cross-link the composite gel, PA was added to the collagen and HA base, the amount of which was equal to the collagen-only sample (Col/HA/PA).

To assess degradation, the obtained sterile hydrogels were incubated in a phosphate buffer (pH 7.2–7.4) at a temperature of 37 °C for 7 days. After 1 week, the stability of hydrogels was investigated.

For SEM analysis the obtained hydrogels were dried at a temperature of 37 °C in a thermostat until constant mass. Before the SEM investigation, the samples were covered with a gold layer with a thickness of 5 nm. This method of sample preparation allowed us to evaluate the native structure and D-periodicity of the formed collagen fibrils.

### 4.2. Kinetics of Collagen Self-Assembly (Turbidity)

The collagen self-assembly dynamics in the presence of phytic acid and hyaluronic acid were characterized by turbidity measurements. Previously, a 2 mg/mL collagen solution was dialyzed against PBS (pH 7.4) using a dialysis bag with a 12–14 kDa cutoff at 4 °C for 48 h. The collagen/PBS solution (pH 7.4) was then transferred to a centrifuge tube to prepare a 0.5 mg/mL collagen solution containing 10% PA. Col/PA systems were transferred to cuvettes (1.5 mL) and self-assembly was initiated by incubation at 37 °C. The self-assembly kinetics of Col/PA (0.1) was continuously monitored by measuring absorbance at 310 nm for up to 70 min using a UV spectrophotometer (UV-2000, UNICO, Shanghai, China).

### 4.3. Scanning Electron Microscopy

The morphology of the surface of collagen gels before/after degradation was assessed using a scanning electron microscope (SEM) JSM-7001F (Jeol, Tokyo, Japan).

### 4.4. FTIR Spectroscopy

The structure of collagen gels before and after adding PA and HA were analyzed by a Fourier transform infrared (FTIR) spectrometer IR Prestige-21 (Shimadzu, Tokyo, Japan) in the transmittance mode in the range of 3200–600 cm^−1^ with a spectral resolution of 4 cm^−1^.

### 4.5. Thermodynamic Properties

The thermal transition was analyzed by the differential scanning calorimetric (DSC) method. The experiment was carried out by using the DSC device (DSC 204 F1, NETZSCH Germany). The scaffolds weighing about 3–4 mg were used and heated from 30 °C to 210 °C at a heating rate of 5 °C min^−1^ in a nitrogen atmosphere. The endothermic peak temperature shifts between the hydrogel were investigated.

To study the temperature of thermal degradation, thermogravimetric analysis (TGA) was performed using the TG 209 F1 Iris device (NETZSCH, Selb, Germany). The sample was heated in an inert medium (argon), ranging from a temperature of 30 °C to 600 °C at a speed of 10 °C/min. The scaffolds weighed about 3–4 mg.

### 4.6. Rheological Properties

Rheological experiments were carried out on the Physica MCR301 rheometric unit (Anton Paar, Graz, Austria GmbH) in the CP25 plane-to-plane measuring system (diameter 25 mm, the gap between the planes was 1 mm) at a temperature of 25 °C. The test was carried out in an oscillating mode in the frequency range from 20 rad/s to 0.1 rad/s. The deformation amplitude was 1%. According to the measurement results, the dependencies G′ (storage modulus) and G″ (loss modulus) on the deformation frequency were constructed.

### 4.7. In Vitro Degradation

The oxyproline assay was used to assess the concentration of collagen in PBS solutions after gel degradation. The gels were incubated in a PBS solution at a temperature of 37 °C. Solution samples were taken for 3–7 days and hydrolyzed in 6 M HCl sealed ampoules at 120 °C for 24 h. The hydrolyzed solution was dried and Chloramine T. was added. The content of released hydroxyproline from the material was measured with ultraviolet spectroscopy at a wavelength of *λ* = 560 nm. The biodegradation degree is defined as the percentage of hydroxyproline released from the samples at different times to the completely degraded one with the same composition and weight. The degradation value for the Col sample was assumed to be 100%. For the remaining samples, degradation was assessed based on this value.

### 4.8. Cell Cultivation

In vitro biocompatibility was also examined using total FetMSCs mesenchymal stem cells from the bone marrow of 5- to 6-week-old human embryos. The cell lines were obtained from the Vertebrate Cell Culture Collection (Institute of Cytology, Russian Academy of Sciences, St. Petersburg, Russia). Cells were cultured in polystyrene flasks in DMEM/F12 medium supplemented with 10% FBS (Fetal Bovine Serum) and 1% penicillin/streptomycin (Sigma-Aldrich, Darmstadt, Germany) at 37 °C in a humidified atmosphere with CO_2_ in the air at 5%. Subconfluent cells were passaged using trypsin-EDTA (0.25% trypsin, 1 mM EDTA).

### 4.9. MTT Assay

Human FetMSCs, HepG2, and HOS cell lines (Institute of Cytology, St. Petersburg) were used to study cytotoxicity. FetMSC cells were cultured in a CO_2_ incubator at 37 °C in a humidified atmosphere containing air and 5% CO_2_ in a DMEM/F12 (for FetMSCs), DMEM (for Hos), MEM (for HepG2) nutrient medium containing 10% (by volume) (Gibco) thermally inactivated fetal bovine serum (FBS; HyClone, St. Louis, MO, USA), 1% L-glutamine, 50 U/mL of penicillin, and 50 mcg/mL of streptomycin. For the experiment, 5.0 × 10^3^ cells/100 µL/well were sown in 96-well plates. A day later, the medium was removed and a nutrient medium with the test substances (PA with different concentrations) was added to the wells on the test substrates and cultured for 72 h. At the end of the incubation period, the medium was removed and 50 µL/well of DMEM/F12 or MEM medium with MTT (0.1 mg/mL) was introduced. The cells were incubated in a CO_2_ incubator for 2 h at 37 °C. After removal of the suprasetting fluid, formazane crystals formed by metabolically viable cells were dissolved in dimethyl sulfoxide (50 µL/well) and transferred to clean wells, and then the optical density was measured at 570 nm on a flatbed spectrophotometer. The analysis of the polynomial regression in Microsoft Excel was used for the calculation.

### 4.10. Fluorescent Staining of Cells

Cells were fluorescently stained with rhodamine phalloidin to study the effect of collagen fibrils before and after hydrogen peroxide treatment on cell adhesion. Pure glass was used as a positive control and unmodified collagen fibrils were used as a negative control.

A precise description of the fluorescent cell staining technique is given in our previously published work [35]. Briefly, staining was performed as follows. At the end of the cultivation period, the medium was removed, and the attached cells were washed with PBS and fixed with a 4% formaldehyde solution (Sigma-Aldrich, St. Louis, MO, USA).

Next, a detergent solution was added to the cells. Rhodamine phalloidin (Thermo Fisher Scientific, Carlsbad, CA, USA) was used to stain the actin and DAPI (ab104139; Abcam, Cambridge, MA, USA) was used to stain the nuclei. The cytoskeleton organization and spreading were analyzed using a confocal microscope Olympus FV3000 (Olympus Corporation, Tokyo, Japan).

### 4.11. Methylene Blue Staining

The change in the shape of the gels was evaluated after staining the samples with 1% methylene blue solution. The gels were previously washed in PBS, fixed in 10% formalin for 30 min, and then stained for 60 s in a methylene blue solution. The methylene blue solution was prepared in a 0.1 M drilling buffer (pH = 8.5, Sigma). After coloring, the gels were washed in water.

### 4.12. Statistical Analysis

All experiments were repeated three to five times. An ANOVA and *t*-test were performed using Microsoft Excel 2019 (ver. 17) software to analyze the statistically significant differences between samples. Data were considered to be statistically important when *p* < 0.05.

## Data Availability

The data presented in this study are openly available in article.

## References

[1] Turnbull G., Clarke J., Picard F., Riches P., Jia L., Han F., Li B., Shu W. (2018). 3D bioactive composite scaffolds for bone tissue engineering. Bioact. Mater..

[2] Nikolova M.P., Chavali M.S. (2019). Recent advances in biomaterials for 3D scaffolds: A review. Bioact. Mater..

[3] Echeverria Molina M.I., Malollari K.G., Komvopoulos K. (2021). Design Challenges in Polymeric Scaffolds for Tissue Engineering. Front. Bioeng. Biotechnol..

[4] El-Sherbiny I.M., Yacoub M.H. (2013). Hydrogel scaffolds for tissue engineering: Progress and challenges. Glob. Cardiol. Sci. Pract..

[5] Sivaraj D., Chen K., Chattopadhyay A., Henn D., Wu W., Noishiki C., Magbual N.J., Mittal S., Mermin-Bunnell A.M., Bonham C.A. (2021). Hydrogel Scaffolds to Deliver Cell Therapies for Wound Healing. Front. Bioeng. Biotechnol..

[6] Gomez-Florit M., Pardo A., Domingues R.M.A., Graça A.L., Babo P.S., Reis R.L., Gomes M.E. (2020). Natural-Based Hydrogels for Tissue Engineering Applications. Molecules.

[7] Xu F., Dawson C., Lamb M., Mueller E., Stefanek E., Akbari M., Hoare T. (2022). Hydrogels for Tissue Engineering: Addressing Key Design Needs Toward Clinical Translation. Front. Bioeng. Biotechnol..

[8] Mantha S., Pillai S., Khayambashi P., Upadhyay A., Zhang Y., Tao O., Pham H.M., Tran S.D. (2019). Smart Hydrogels in Tissue Engineering and Regenerative Medicine. Materials.

[9] Davari N., Bakhtiary N., Khajehmohammadi M., Sarkari S., Tolabi H., Ghorbani F., Ghalandari B. (2022). Protein-Based Hydrogels: Promising Materials for Tissue Engineering. Polymers.

[10] Zhang Y., Wang Y., Li Y., Yang Y., Jin M., Lin X., Zhuang Z., Guo K., Zhang T., Tan W. (2023). Application of Collagen-Based Hydrogel in Skin Wound Healing. Gels.

[11] Sarrigiannidis S.O., Rey J.M., Dobre O., González-García C., Dalby M.J., Salmeron-Sanchez M. (2021). A tough act to follow: Collagen hydrogel modifications to improve mechanical and growth factor loading capabilities. Mater. Today Bio.

[12] Gurumurthy B., Bierdeman P.C., Janorkar A.V. (2016). Composition of elastin like polypeptide–collagen composite scaffold influences in vitro osteogenic activity of human adipose derived stem cells. Dent. Mater..

[13] Lee J.H., Lee J.-Y., Yang S.H., Lee E.-J., Kim H.-W. (2014). Carbon nanotube–collagen three-dimensional culture of mesenchymal stem cells promotes expression of neural phenotypes and secretion of neurotrophic factors. Acta Biomater..

[14] Park J.W., Kang Y.D., Kim J.S., Lee J.H., Kim H.-W. (2014). 3D microenvironment of collagen hydrogel enhances the release of neurotrophic factors from human umbilical cord blood cells and stimulates the neurite outgrowth of human neural precursor cells. Biochem. Biophys. Res. Commun..

[15] Entekhabi E., Haghbin Nazarpak M., Shafieian M., Mohammadi H., Firouzi M., Hassannejad Z. (2021). Fabrication and in vitro evaluation of 3D composite scaffold based on collagen/hyaluronic acid sponge and electrospun polycaprolactone nanofibers for peripheral nerve regeneration. J. Biomed. Mater. Res.

[16] Ying H., Zhou J., Wang M., Su D., Ma Q., Lv G., Chen J. (2019). In situ formed collagen-hyaluronic acid hydrogel as biomimetic dressing for promoting spontaneous wound healing. Mater. Sci. Eng. C.

[17] Vázquez-Portalatín N., Kilmer C.E., Panitch A., Liu J.C. (2016). Characterization of Collagen Type I and II Blended Hydrogels for Articular Cartilage Tissue Engineering. Biomacromolecules.

[18] Tsaryk R., Gloria A., Russo T., Anspach L., De Santis R., Ghanaati S., Unger R.E., Ambrosio L., Kirkpatrick C.J. (2015). Collagen-low molecular weight hyaluronic acid semi-interpenetrating network loaded with gelatin microspheres for cell and growth factor delivery for nucleus pulposus regeneration. Acta Biomater..

[19] Xin X., Borzacchiello A., Netti P.A., Ambrosio L., Nicolais L. (2004). Hyaluronic-acid-based semi-interpenetrating materials. J. Biomater. Sci. Polym. Ed..

[20] Avendano A., Chang J.J., Cortes-Medina M.G., Seibel A.J., Admasu B.R., Boutelle C.M., Bushman A.R., Garg A.A., DeShetler C.M., Cole S.L. (2020). Integrated Biophysical Characterization of Fibrillar Collagen-Based Hydrogels. ACS Biomater. Sci. Eng..

[21] Lai V.K., Nedrelow D.S., Lake S.P., Kim B., Weiss E.M., Tranquillo R.T., Barocas V.H. (2016). Swelling of Collagen-Hyaluronic Acid Co-Gels: An In Vitro Residual Stress Model. Ann. Biomed. Eng..

[22] Nashchekina Y.A., Sirotkina M.Y., Darvish D.M., Barsuk I.A., Moskalyuk O.A., Mikhailova N.A. (2021). The Effect of Carbodiimide on the Structural, Mechanical and Biological Properties of Collagen Films. Cell Tiss. Biol..

[23] Kontturi L.-S., Järvinen E., Muhonen V., Collin E.C., Pandit A.S., Kiviranta I., Yliperttula M., Urtti A. (2014). An injectable, in situ forming type II collagen/hyaluronic acid hydrogel vehicle for chondrocyte delivery in cartilage tissue engineering. Drug Deliv. Transl. Res..

[24] Heo J., Koh R.H., Shim W., Kim H.D., Yim H.-G., Hwang N.S. (2016). Riboflavin-induced photo-crosslinking of collagen hydrogel and its application in meniscus tissue engineering. Drug Deliv. Transl. Res..

[25] Forgione D., Nassar M., Seseogullari-Dirihan R., Jamleh A., Tezvergil-Mutluay A. (2023). Effect of phytic acid on dentinal collagen solubilization and its binding and debinding potentials to dentin. J. Dent..

[26] Ravichandran R., Seitz V., Reddy Venugopal J., Sridhar R., Sundarrajan S., Mukherjee S., Wintermantel E., Ramakrishna S. (2013). Mimicking Native Extracellular Matrix with Phytic Acid-Crosslinked Protein Nanofibers for Cardiac Tissue Engineering. Macromol. Biosci..

[27] Dost K., Tokul O. (2006). Determination of phytic acid in wheat and wheat products by reverse phase high performance liquid chromatography. Anal. Chim. Acta.

[28] Febles C.I., Arias A., Hardisson A., Rodríguez-Alvarez C., Sierra A. (2002). Phytic Acid Level in Wheat Flours. J. Cereal. Sci..

[29] Forgione D., Nassar M., Seseogullari-Dirihan R., Thitthaweerat S., Tezvergil-Mutluay A. (2021). The effect of phytic acid on enzymatic degradation of dentin. Eur. J. Oral Sci..

[30] Wang X., Wen K., Yang X., Li L., Yu X. (2017). Biocompatibility and anti-calcification of a biological artery immobilized with naturally-occurring phytic acid as the crosslinking agent. J. Mater. Chem. B.

[31] Tu X., Chen X., Peng Y., Nan J., Wei B., He L., Xu C., Xu Y., Xie D., Zhang J. (2018). Modulation of the Self-Assembly of Collagen by Phytic Acid: An In Vitro Study. Macromol. Res..

[32] Espinosa-Andrews H., Velásquez-Ordoñez C., Cervantes-Uc J.M., Rodríguez-Rodríguez R. (2023). Water behavior, thermal, structural, and viscoelastic properties of physically cross-linked chitosan hydrogels produced by NaHCO3 as a crosslinking agent. J. Mater. Sci..

[33] Noitup P., Morrissey M.T., Garnjanagoonchorn W. (2006). In vitro self-assembly of silver-line grunt type I collagen: Effects of collagen concentrations, pH and temperatures on collagen self-assembly. J. Food Biochem..

[34] Ficai A., Andronescu E., Voicu G., Ghitulica C., Vasile B.S., Ficai D., Trandafir V. (2010). Self-Assembled Collagen/Hydroxyapatite Composite Materials. Chem. Eng. J..

[35] Nashchekina Y., Nikonov P., Mikhailova N., Nashchekin A. (2021). Collagen scaffolds treated by hydrogen peroxide for cell cultivation. Polymer.

[36] Fassett J., Tobolt D., Hansen L.K. (2006). Type I Collagen Structure Regulates Cell Morphology and EGF Signaling in Primary Rat Hepatocytes through CAMP-Dependent Protein Kinase A. Mol. Biol. Cell.

[37] Nashchekina Y.A., Starostina A.A., Trusova N.A., Sirotkina M.Y., Lihachev A.I., Nashchekin A.V. (2020). Molecular and fibrillar structure collagen analysis by FTIR spectroscopy. J. Phys. Conf. Ser..

[38] Muyonga J.H., Cole C.G.B., Duodu K.G. (2004). Characterisation of Acid Soluble Collagen from Skins of Young and Adult Nile Perch (Lates Niloticus). Food Chem..

[39] Lv Q., Hu K., Feng Q., Cui F. (2008). Fibroin/Collagen Hybrid Hydrogels with Crosslinking Method: Preparation, Properties, and Cytocompatibility. J. Biomed. Mater. Res. A.

[40] Demeter M., Călina I., Scărișoreanu A., Micutz M., Kaya M.A. (2022). Correlations on the Structure and Properties of Collagen Hydrogels Produced by E-Beam Crosslinking. Materials.

[41] Zhang Y., Liu W., Li G., Shi B., Miao Y., Wu X. (2007). Isolation and partial characterization of pepsin-soluble collagen from the skin of grass carp (*Ctenopharyngodon idella*). Food Chem..

[42] Collins M.N., Birkinshaw C. (2008). Physical properties of crosslinked hyaluronic acid hydrogels. J. Mater. Sci. Mater. Med..

[43] Daneluti A.L.M., Velasco M.V.R., Baby A.R., Matos J.D.R. (2015). Thermal behavior and free-radical-scavenging activity of phytic acid alone and incorporated in cosmetic emulsions. Cosmetics.

[44] Velegol D., Lanni F. (2001). Cell traction forces on soft biomaterials. I. Microrheology of Type I collagen gels. Biophys. J..

[45] Tytgat L., Markovic M., Qazi T.H., Vagenende M., Bray F., Martins J.C., Rolando C., Thienpont H., Ottevaere H., Ovsianikov A. (2019). Photo-crosslinkable recombinant collagen mimics for tissue engineering applications. J. Mater. Chem. B.

[46] Horn M.M., Martins V.C.A., de Guzzi Plepis A.M. (2009). Interaction of anionic collagen with chitosan: Effect on thermal and morphological characteristics. Carbohydr. Polym..

[47] Fratzl P. (2008). Collagen: Structure and Mechanics.

[48] Ding C., Zhang M., Tian H., Li G. (2013). Effect of hydroxypropyl methylcellulose on collagen fibril formation in vitro. Int. J. Biol. Macromol..

[49] Cheng X., Gurkan U.A., Dehen C.J., Tate M.P., Hillhouse H.W., Simpson G.J., Akkus O. (2008). An Electrochemical Fabrication Process for the Assembly of Anisotropically Oriented Collagen Bundles. Biomaterials.

[50] Li Y., Asadi A., Margo R.M., Elliot P.D. (2009). pH effects on collagen fibrillogenesis *in vitro*: Electrostatic interactions and phosphate binding. Mater. Sci. Eng. C-Mater..

[51] Tian H., Li C., Liu W., Li J., Li G. (2013). The influence of chondroitin 4-sulfate on the reconstitution of collagen fibrils in vitro. Colloid Surface B.

[52] Lou J., Stowers R., Nam S., Xia Y., Chaudhuri O. (2018). Stress relaxing hyaluronic acid-collagen hydrogels promote cell spreading, fiber remodeling, and focal adhesion formation in 3D cell culture. Biomaterials.

[53] Sato N., Taniguchi T., Goda Y., Kosaka H., Higashino K., Sakai T., Katoh S., Yasui N., Sairyo K., Taniguchi H. (2016). Proteomic Analysis of Human Tendon and Ligament: Solubilization and Analysis of Insoluble Extracellular Matrix in Connective Tissues. J. Proteome Res..

[54] Frantz C., Stewart K.M., Weaver V.M. (2010). The extracellular matrix at a glance. J. Cell Sci..

[55] Bancroft J.D., Suvarna S.K., Layton C. (1992). The Theory and Practice of Histological Techniques.

[56] Kiani C., Chen L., Wu Y.J., Yee A.J., Yang B.B. (2002). Structure and function of aggrecan. Cell Res..

[57] Solis M.A., Chen Y.H., Wong T.Y., Bittencourt V.Z., Lin Y.C., Huang L.L.H. (2012). Hyaluronan regulates cell behavior: A potential niche matrix for stem cells. Biochem. Res. Int..

[58] Slaughter B.V., Khurshid S.S., Fisher O.Z., Khademhosseini A., Peppas N.A. (2009). Hydrogels in regenerative medicine. Adv. Mater..

[59] Nashchekina Y.A., Yudintceva N.M., Nikonov P.O., Ivanova E.A., Smagina L.V., Voronkina I.V. (2017). Effect of Concentration of Collagen Gel on Functional Activity of Bone Marrow Mesenchymal Stromal Cell. Bull. Exp. Biol. Med..

